# An examination of the initial cancer consultation of medical and radiation oncologists using the Cancode interaction analysis system

**DOI:** 10.1038/sj.bjc.6604348

**Published:** 2008-04-29

**Authors:** A Dimoska, P N Butow, E Dent, B Arnold, R F Brown, M H N Tattersall

**Affiliations:** 1 Medical Psychology Research Unit, Faculty of Medicine, Blackburn Building (Do6), University of Sydney, Sydney, New South Wales 2006, Australia; 2 School of Psychology, Brennan MacCallum Building (A18), University of Sydney, Sydney, New South Wales 2006, Australia; 3 Institute for Doctor–Patient Communication, Department of Medicine, University of Pittsburgh, PA 15260, USA; 4 Department of Psychiatry and Behavioral Sciences, Memorial Sloan-Kettering Cancer Center, 641 Lexington Avenue, New York, NY 10025, USA; 5 Department of Cancer Medicine, Blackburn Building (D06), University of Sydney, Sydney, New South Wales 2006, Australia

**Keywords:** oncology, consultation, communication, interaction analysis

## Abstract

This study provides an analysis of the structure of the initial cancer consultation, the consultation styles of medical and radiation oncologists, and their effect on patient outcomes. One hundred and fifty-five cancer patients attending their first consultation with either a medical or radiation oncologist were audiotaped and the transcripts were analysed using the Cancode computer interaction analysis system. Findings revealed that medical oncologists allowed patients and their families more input into the consultation and were rated as warmer and more patient-centred compared with radiation oncologists. However, radiation oncologists spent a longer period discussing, and were more likely to bring up, social support issues with patients. Both medical and radiation oncologists varied their consultation style according to the patient's gender, age, anxiety levels, prognosis, and education. Patients seeing an oncologist who was rated as warmer and discussed a greater number of psychosocial issues had better psychological adjustment and reduced anxiety after consultation. These findings provide current evidence that may be used to inform improvements of communication skills training for oncologists and highlight the need for future communication research to separately consider oncologists from different disciplines.

The most widely recommended model of medical interactions in clinical practise is patient-centred care. A patient-centred approach is one in which the doctor listens to patients attentively and sympathetically, talks about psychosocial and non-medical issues (Arora, 2003), appears warm and caring towards the patient rather than hurried, and allows the patient to have input into the consultation (Butow *et al*, 1995). Researchers have identified the importance of doctors varying their consultation style in response to differing patient characteristics (Butow *et al*, 1995). For example, researchers suggest that doctors should match their style to patient preferences for involvement in decision-making (Keisler and Auerbach, 2006), and recommend responding flexibly to patients' emotional and informational cues (Butow *et al*, 2002). Others have found that male and female patients may benefit from different communication strategies (Butow *et al*, 1997; Parker *et al*, 2001).

Many studies have shown an association between patients receiving patient-centred care in their consultation and subsequent positive patient outcomes (Fogarty *et al*, 1999). However, previous studies have suggested that patient-centred care and flexibility in consultation style are used inconsistently in medical consultations. Oncologists have been shown to be poor judges of patient preferences for participation, significantly underestimating cancer patients' preference for a shared approach to decision-making (Bruera *et al*, 2002) and desire for information. They also tend to overestimate the amount of information they believe they have given (Chaitchik *et al*, 1992) and cancer patients' understanding of this information (Gattellari *et al*, 1999). On the other hand, in one of our earlier studies, an interaction analysis of 142 consultations of cancer patients seeing a male medical oncologist, we found evidence of flexibility (Butow *et al*, 1995). The oncologist was more affiliative with anxious patients and females, spent more time answering the questions of patients who asked more questions, and spoke for a longer period with younger patients about prognostic and treatment issues.

One source of variation in patient-centred care may be doctor characteristics. Female doctors have been shown to use a more patient-centred communication style than male doctors (for a review see Roter *et al*, 2002). Another source of variation may be the medical context, such as specialty or discipline. Brown *et al* (2001) found evidence that the consultations of radiation oncologists tended to be shorter than the consultations of medical oncologists. However, little is known about the differences in consultation style and structure between oncologists from various disciplines and their concurrent effects on patient behaviour and outcomes.

The aims of this study were to examine the structure of cancer consultations in a larger sample of medical oncologists and to provide a comparison with the consultations of radiation oncologists using the Cancode interaction analysis system (Dent *et al*, 2005). Although the examination between medical and radiation oncologists is exploratory, across groups we anticipate that

1. medical and radiation oncologists will vary their consultation style and duration of talk in accord with the demographic and disease characteristics of the patient, as well as with their levels of anxiety and information preferences; and
2. patient satisfaction, information recall, and psychological adjustment will be higher when the consultation is characterised by a patient-centred approach.

## MATERIALS AND METHODS

Cancer patients attending their first outpatient consultation with one of five medical oncologists and four radiation oncologists at one of two university teaching hospitals in Sydney were invited to participate in this study once consent had been given by the oncologist. Patients were excluded on the following criteria: (i) age less than 16 years, (ii) non-English speaking, (iii) advanced incapacity, and (iv) unavailability for the duration of follow-up. Ten per cent of patients declined to participate, resulting in a sample of 155 patients. Five medical oncologists ranging in age from 39 to 58 years (mean 47.9 years) and four radiation oncologists aged 37–42 years (mean 38.3 years) were invited to participate and agreed. The ratio of female to male oncologists was 1 : 4 and 2 : 2 for medical and radiation oncologists, respectively.

### Procedure

Before their consultation, patients were informed of the purpose and procedures of the study. Written permission was obtained for their participation and to audiotape the consultation. Patients then completed questionnaires measuring anxiety, and preferences for information and involvement in decision-making. Immediately after the consultation, anxiety was measured again, and 7–10 days after the consultation, patients were mailed questionnaires assessing anxiety, preferences for information, and involvement in decision-making, satisfaction, and psychological adjustment to cancer. The project received ethical approval from the Central Sydney Area Health Service and the University of Sydney Ethics Committees.

### Coding

The consultations were transcribed by three experienced researchers familiar with medical terminology and periodically checked for accuracy. Subsequently, two coders who were trained in applying the Cancode manual used the standard procedure of listening to audiotapes while reading and marking codes directly onto the transcripts. Coders re-coded a random 10% of their own consultations and 10% of the other's consultations. This process is outlined by Brown *et al* (2001). In addition to coding of transcripts, coders provided subjective ratings using visual analogue scales (ranging 0–10) on different aspects of the overall consultation style, including the competence of the doctor, clarity of information delivery, whether they were hurried, patient- or doctor-centered, and whether they responded warmly or not to the patient, as well as the emotional valence (+/−) of the message. Coders made a macrolevel subjective assessment for each of the dimensions and marked this at a point along a 100-mm line with descriptors at each extreme. Scores were obtained by measuring the distance in centimeters from the left extreme (0 point).

### Cancode interaction analysis

Cancode was adapted from CN-LOGIT and consists of three parts: (1) micro-level analysis in real time, retaining the sequence of events, (2) event counts, and (3) macro-level analysis of consultation style and effect. The consultation is divided into units of speech, which change when a person stops speaking or changes speech content, and each unit is classified along four dimensions (source, content, function, and emotion) (see Dent *et al*, 2005 for description of units within each dimension). The coder enters the codes by keyboard into a specifically designed software package while listening to the audiotape in real time. The space bar marks the end of a speech segment. The software calculates the duration of time and frequency (i.e., count) for each individual code and combination of codes, as well as the duration of the total consultation. Thus, the data events are summed into higher order categories (e.g., duration of all diagnosis events across function types). The resultant data sheet is automatically converted into a spreadsheet in the Statistical Package for the Social Sciences (SPSS) for further analysis. The reliability and validity of the CANCODE system has been previously established. Inter-rater reliability was 0.50 for function and 0.59 for content, whereas intra-rater reliability was 0.86 for content and 0.80 for function (Dent *et al*, 2005). In this study, based on the 10% of transcripts checked, our coders obtained a high intra-rater reliability of 0.95 for the content category and an inter-rater reliability of 0.92.

### Measures

Demographic characteristics were obtained from patients and oncologists by a research assistant, whereas disease characteristics and treatment goals were obtained from oncologists about each patient.

Anxiety was measured using the Speilberger State Anxiety Scale (SSAS) (Speilberger, 1983), which is widely used for measuring situational anxiety. Patient satisfaction was measured using the 25 items adapted from Roter (1977) and Korsch *et al* (1968). Items addressed satisfaction with the amount and quality of information received (e.g., ‘The doctor explained my condition clearly’), the doctor's communication skills (e.g., ‘The doctor sometimes interrupted me’), and the patients' participation in the consultation (e.g., ‘I asked all the questions I wanted to’). Responses were on a 5-point Likert scale ranging from ‘I disagree completely’ to ‘I agree completely’. All satisfaction scores were converted to percentages of the maximum possible score.

Recall of information by patients was assessed using a structured telephone interview. Firstly, information obtained from transcripts was categorised into one of five categories, giving an estimate of the number and type of ‘facts’ potentially available to each patient for recall. Patients were asked an open question about ‘what the doctor said’ (spontaneous recall) followed by standardised prompts (prompted recall) covering the five categories. Each item recalled was compared with the specific information presented by oncologists, and spontaneous and prompted recall were summed to give a total recall figure, which was then reported as the percentage of facts recalled accurately of the total number of facts across categories. A fuller account of this process can be found in Dunn *et al* (1993).

Psychological adjustment was measured using the Mental Adjustment to Cancer Scale (MAC) (Watson *et al*, 1988), which consists of five subscales including Fighting Spirit, Helpless/Hopeless, Anxious Preoccupation, Fatalism, and Denial (Avoidance).

Information and involvement preferences included (a) general information preferences measured by two items from the Cassileth Information Styles Questionnaire (Cassileth *et al*, 1980): (1) the amount of detail required (5-point Likert scale) and (2) the type of information (‘only sufficient to care for myself’, ‘only good news’, or ‘all news’); (b) preferred level of involvement in decision-making measured using a scale developed by Sutherland *et al* (1989) (five categories ranging from ‘patient only’ to ‘doctor only’ making decisions); and (c) specific information and support preferences using 12 items adapted from the Cassileth Information Styles Questionnaire (Cassileth *et al*, 1980).

Ratios were calculated from coded events and included (1) the ratio of speaking time by doctors compared with patients, calculated by dividing the total duration of doctor speech events by the total duration of patient speech events, and (2) the ratio of speech events dedicated to psychosocial issues was calculated as the frequency of total speech events (across doctor and patient) for psychosocial/social support issues divided by the frequency of biomedical issues discussed (i.e., history/symptoms, diagnosis, prognosis, treatment, and other medical categories).

### Statistical analysis

Multiple and univariate linear regressions analyses and ANOVAs with planned comparisons were used to explore the effects of demographic, disease, and consultation variables on the outcome measures. Univariate analyses examined the variability of oncologists' behaviour with patient characteristics, across medical and radiation specialty in the first instance. Subsequently, multiple regression analyses were modelled with the following factors: specialty group (medical *vs* radiation), patient age, gender, stage and type of disease, prognosis, information and involvement preferences, decision-making preference, anxiety, the presence/absence of family members, marital status, education level, occupation, and length of illness. There was insufficient variability in information preference (13 out of 154 patients wanted less than all news) and in detail (16 patients wanted ‘some’ information and 17 patients wanted ‘a lot of’ information) to model these variables meaningfully.

## RESULTS

One hundred and fifty-five patients completed the anxiety and information/involvement preferences questionnaires and had their consultations recorded. Table 1 shows the demographic and clinical characteristics of patients. Eighty-one patients saw a medical oncologist (29% with a family member) and 73 patients saw a radiation oncologist (49% with a family member). Of these, 133 (86%) returned the questionnaires (2 did not complete the anxiety questionnaire) that were posted out 1-week after their consultation and 126 (82%) completed the follow-up interview. There were no significant differences between patients retained and patients lost to follow-up, suggesting that there was no apparent bias in the study sample.

### Structure of consultations

#### Factors influencing consultation duration

**Figure 1:**
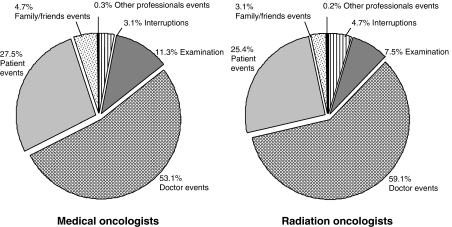
Different ‘sources’ of consultation events shown as a proportion of the total duration of the consultation.

Overall consultation duration was longer for medical than radiation oncologists (36.7 *vs* 23.1 min, F(1, 153)=60.2, *P*<0.001), and this was due to longer physical examinations (4.3 *vs* 1.6 min, F(1, 153)=40.8, *P*<0.001), the greater length of time the doctor spoke (18.5 *vs* 13.4 min, F(1, 152)=22.0, *P*<0.001), as well as allowing more time for the patient (10.6 *vs* 6.2 min, F(1, 152)=29.5, *P*<0.001) and the family to speak (1.9 *vs* 1.6 min, F(1, 152)=16.0, *P*<0.001). Interruptions took up 3.1% of the consultation for medical oncologists and 4.7% for radiation oncologists. Figure 1 shows different ‘sources’ of consultation events shown as a proportion of the total duration of the consultation.

### Duration of consultation categories

Table 2 details the duration for each content category for medical and radiation oncologist consultations. Treatment was spoken about for the longest period of time, followed by history and symptoms, diagnosis, and then prognosis. Compared with radiation oncologists, medical oncologists spoke longer to patients about history and symptoms, diagnosis, prognosis, and treatment. Radiation oncologists spoke longer about social support/counselling/stress management; however, it should be noted that this topic was brought up in only 13.2% of radiation oncology consultations. Five per cent of medical oncology consultations involved discussion about support issues. Prognosis was not brought up at all in 5.7 and 8.3% of medical and radiation oncology consultations, respectively. Psychosocial issues were not discussed by medical oncologists in 33.3% of consultations and by radiation oncologists in 25% of consultations, and there was no social exchange initiated by doctors in 17.2 and 20.6% of consultations for medical and radiation oncologists, respectively.

Table 3 shows the breakdown of time spent for each function of speech events for medical and radiation oncologists and their patients. Medical oncologists made on average 37.6 informative/educational statements, with half of these about treatment (47% of statements made), and asked 46.6 questions primarily about history and symptoms. Radiation oncologists made a similar number of informative/educational statements (38.5) with half of these about treatment, but asked significantly less questions (28.5) (F(1, 153)=31.5, *P*<0.001). Medical oncologists spent more time informing or educating the patient (medical 11.1 min *vs* radiation 8.9 min, F(1, 153)=6.1, *P*<0.05). However, very little time was spent on checking patient understanding with an average of 11 s (<1% of total patient+doctor speaking time), or on building rapport (partnership building and active support) with an average of 31 s (2.1% of the total speaking time), and there were no differences between medical and radiation oncologists. Therefore, it is not surprising to see that patients spent an average of only 12 s (<1% of the consultation) expressing feelings or seeking reassurance.

Patients seeing a medical oncologist made a greater number of informative statements compared with patients seeing a radiation oncologist (61 *vs* 41, F(1, 153)=22.2, *P*<0.001), but the number of questions asked between medical (12.1) and radiation (10.4) oncology patients was similar. Half the questions in both groups were about treatment. Patients who asked a greater number of questions overall were younger (13.1 *vs* 9.7 (older); F(1, 151)=5.4, *P*<0.05), female (12.8 *vs* 10.1 (male); F(1, 153)=3.3, *P*=0.072), and had a higher education level (<high school 7.4 *vs* high school 11.6 *vs* tertiary 15.0; F(1, 150)=8.6, *P*<0.001).

### Ratio of doctor to patient talk

The ratio of doctor to patient talk ranged from 0.5 to 11.2 for medical oncologists (mean 2.2) and 0.07–45.4 for radiation oncologists (mean 4.1), and the difference between groups was significant (F(1, 153)=6.4, *P*<0.05). Medical oncologists also discussed a greater number of points (117 *vs* 96 points, F(1, 153)=10.5, *P*<0.01) and spent a longer period discussing each point compared with radiation oncologists (9.7 *vs* 8.7 s, F(1, 153)=4.6, *P*<0.05). However, the average ratio of the number of times psychosocial/social support issues was discussed between patients and oncologists, relative to biomedical issues, was greater for radiation than medical oncologists (0.06 *vs* 0.14, F(1, 153)=14, *P*<0.001).

### Global ratings of doctor behaviour

On 10-point visual analogue scales, medical oncologists were rated as more competent and confident, better at communicating information clearly to the patient, more patient-centred in their consultation style, and less hurried in the consultation compared with radiation oncologists. See Table 4 for a list of items on consultation style, mean scores of global ratings, and statistical results.

### Variation in doctor behaviour with patient characteristics

#### Consultation style

Univariate analyses showed that whether oncologists were patient-centred, relaxed and warm was independent of patient anxiety, stage of disease, length of illness, marital status, occupation, treatment decision preferences, or whether or not a family member was present. However, oncologists were more patient-centred with female patients (*t*_141_=3.0, *P*<0.01) and tended to be more patient-centred with patients who had a long prognosis (i.e., normal life expectancy *vs* weeks to months)(*t*_126_=3.5, *P*=0.063). Oncologists were also warmer with female patients (*t*_142_=−2.2, *P*<0.05) and tended to be warmer with patients who had a long prognosis (*t*_127_=1.8, *P*=0.076). Oncologists were more relaxed with younger patients (*t*_141_=−3.0, *P*<0.01) and this effect remained significant even after controlling for doctor age.

In a multiple regression examining factors associated with oncologists' patient-centredness, gender remained significant (*t*_138_=2.2, *P*<0.05) and decision-making preferences (*t*_138_=−3.2, *P*<0.01) became significant. This latter effect revealed that doctor-centred care was given to patients who wanted their doctor to make their treatment decision for them and patient-centred care was given to patients who wanted to make their own treatment decision. With regard to whether doctors were warm or cold, gender remained significant (*t*_126_=−2.0, *P*<0.05) and prognosis tended towards significance (*t*_126_=1.8, *P*=0.079). Patient age was still the only variable that predicted whether the doctor was relaxed or not (*t*_138_=−3.0, *P*<0.01). Specialty group did not interact with any factors.

### Duration of time doctor spoke

Univariate analyses revealed that oncologists spoke longer with younger patients (*t*_150_=−2.9, *P*<0.01), female patients (*t*_153_=−2.5, *P*<0.05), patients who were more anxious before their consultation (*t*_152_=3.1, *P*<0.01), patients who preferred to make their own treatment decision (*t*_152_=3.9, *P*<0.001), patients with higher education (*t*_151_=5.0, *P*<0.001), patients who had an occupation as a professional (*t*_145_=−3.8, *P*<0.001), and when there was a family member present (*t*_153_=3.2, *P*<0.01).

Multivariate analyses showed that patient gender (*t*_140_=−3.3, *P*<0.01), patient anxiety (*t*_140_=2.5, *P*<0.05), and patient education level (*t*_140_=4.0, *P*<0.001) influenced the duration that the oncologist spoke for, whereas patient age (*t*_140_=−2.0, *P*=0.052) and whether a family member was present (*t*_140_=1.8, *P*=0.076) approached significance. Furthermore, patient age interacted with gender (*t*_140_=2.5, *P*<0.05). Oncologists talked longer with younger than older patients (median-split) when they were female (18.5 *vs* 12.8 min, F(1, 66)=9.2, *P*<0.01), whereas there was no difference between younger and older male patients (14.0 *vs* 12.2 min, F(1, 82)=2.2, *P*=0.138). The total time oncologists spent speaking, as a proportion of the whole consultation, did not differ with age or gender (*P*>0.10). Specialty group did not interact with any factors.

### Influence of doctor behaviour on patient outcomes

#### Patient anxiety, recall, and satisfaction

Reduced patient anxiety immediately after consultation (Pre-consultation anxiety is subtracted from post-consultation measures of anxiety) was associated with a doctor who was more warm (*t*_140_=−3.2, *P*<0.01) and spoke longer about psychosocial issues (*t*_151_=−2.7, *P*<0.01). Reduced anxiety 1 week post-consultation was associated with a doctor who was more warm (*t*_125_=−2.2, *P*<0.05), and tended to be associated with a doctor who was more relaxed (*t*_125_=−1.9, *P*=0.062) and spoke longer about psychosocial issues (*t*_136_=−1.8, *P*=0.082). Greater patient information recall was associated with shorter consultations (*t*_123_=−4.1, *P*<0.001). Patients seeing a radiation oncologist were more satisfied when the psychosocial to biomedical ratio was greater; however, patients seeing a medical oncologist did not show the same effect (F(1, 129)=15.4, *P*<0.001).

#### Psychological adjustment

Lower scores on the helpless/hopeless scale (‘I feel like giving up’) were associated with a warmer doctor (*t*_119_=−2.0, *P*=0.051) and a shorter consultation (*t*_130_=2.8, *P*<0.01). Lower anxious preoccupation (‘It is a devastating feeling’) was associated with a shorter consultation (*t*_130_=3.5, *P*<0.01) and a higher psychosocial to biomedical ratio (*t*_130_=−2.3, *P*<0.05). Reduced avoidance (‘I deliberately push all thoughts of cancer out of my mind’) was associated with a shorter consultation (*t*_130_=3.4, *P*<0.01). Fighting spirit (‘I see my illness as a challenge’) and fatalism (‘I've put myself in the hands of God’) did not vary. Patients seeing a medical oncologist showed larger scores on the helpless/hopeless (F(1, 130)=7.1, *P*<0.01), anxious preoccupation (F(1, 130)=12.3, *P*<0.01) and avoidance scales (F(1, 130)=4.5, *P*<0.05), compared with radiation oncologists. However, effects relating to specialty and consultation duration did not remain significant when patient anxiety pre-consultation was accounted for (*P*>0.10), as patients were more anxious before medical (which were also longer) than radiation oncology consultations (F(1, 153)=4.1, *P*<0.05).

## DISCUSSION

The Cancode interaction analysis system allowed a fine-grained breakdown of the cancer consultation in this study in terms of frequency and duration of content areas and forms of language. Overall, medical oncologists appeared to adopt a more patient-centred approach than radiation oncologists. They were rated as warmer and less hurried in their consultation style, and allowed the patient to have more input into the consultation. Specifically, medical oncologists spoke longer about history and symptoms, diagnosis, prognosis and treatment, and asked patients more questions. Radiation oncologists appeared to dominate the consultation speaking on average four times longer than their patients, compared with medical oncologists who spoke twice as long as their patients. However, radiation oncologists spent a longer period discussing social support, counselling and stress management, and were more likely than medical oncologists to bring these issues up in the consultation.

These specialty differences may be due to a number of factors. Patients referred for a radiation oncology opinion are usually well informed about the reason for considering radiotherapy, whether as a surgical adjuvant or to treat localised symptoms due to cancer. Consequently, the discussion with the radiation oncologist about antecedent history and treatment will be limited, and the major topic of discussion will be about the treatment, which will commonly last a maximum of 6 weeks, and often less. In comparison, the discussion of risk benefit of chemotherapy and factors influencing absolute risk will commonly be informed by a more detailed antecedent history, assessment of co-morbidities, and consideration of the patient's attitude to uncertainty. Chemotherapy discussions will commonly extend over several months, and the discussion about the morbidity of treatment may be more extensive.

Many studies have identified that oncologists often provide patients with little psychosocial support in consultations (for review see Arora, 2003), and this was found to be true in this study also with an average of 34 and 43 s dedicated to this area in medical and radiation oncology consultations, respectively. Although it is worthwhile noting that in one study, 40 s of compassion was enough to significantly reduce anxiety in breast cancer patients (Fogarty *et al*, 1999). Therefore, although the time devoted to psychosocial support was brief, it may be that this was adequate for some patients. Nevertheless, the ratio of the number of psychosocial to biomedical issues discussed was low at 0.14, and similar to the 0.2 ratio reported by Ford *et al* (1996), indicating that 8.3 biomedical issues were discussed for every one psychosocial issue. Similar to Gattellari *et al* (1999), we also found that oncologists spent little time checking patient understanding (<1% of the consultation) despite spending over half their time informing or educating the patient. However, discussions involving prognosis, which has long been identified as a stressful task for oncologists (Ptacek *et al*, 1999), were only avoided in 5.7 and 8.3% of medical and radiation consultations, respectively. In Butow *et al* (1995), this topic was not brought up in 33% of consultations examined, indicating a clear improvement over the years in oncologists addressing prognostic issues.

As hypothesised, doctors varied their consultation style with patient gender, age, and the prognosis of the patient. This did not differ between medical and radiation oncologists. Overall, oncologists were more patient-centred and warmer towards female than male patients, and they were more relaxed with younger than older patients. Oncologists also spoke for longer periods with younger than older patients, particularly when they were female, and with patients who had a higher education level and when a family member was present. This was in response to these patients asking more questions. These findings are in line with our earlier study (Butow *et al*, 1995) and suggest that oncologists responded to patients' needs for information accordingly. However, a disconcerting finding was that oncologists showed a small trend towards being more patient-centred and warmer towards patients with a normal life expectancy than patients with a prognosis of weeks to months. This finding highlights the need for personal reflection and consultation skills training of oncologists to assist them to cope, and be able to deal more effectively with, patients who have a shorter expected prognosis. Nevertheless, oncologists were shown to respond well to patients' behavioural cues of anxiety, as they spent a greater amount of time talking to patients who reported being more anxious before the consultation.

Patient outcomes were significantly affected by whether doctors adopted a patient-centred approach or not, in line with past findings (Fogarty *et al*, 1999). Although this is in contrast to our earlier study (Butow *et al*, 1995), a greater number of oncologists were included in this study. For example, patient anxiety may be alleviated and patient satisfaction improved by increasing psychosocial discussion. This for the oncologist involves providing more information about the impact the cancer will have on the patient's lifestyle and about available support services. A ‘warm’ and ‘relaxed’ oncologist is also likely to alleviate patient anxiety and may help to improve psychological adjustment. Although appearing ‘warm’ is based on the patient's subjective judgment, it may be as simple as acknowledging any emotions that the patient is expressing, exercising reflecting listening, and providing empathetic responses. Better information recall was associated with a shorter consultation, suggesting that fewer facts may have been presented, which were easier to recall at a later time. However, this effect should not replace the requirement to fully satisfy the informational needs of patients. Furthermore, patients also showed better psychological adjustment after a shorter consultation, although these effects were accounted for by patient anxiety before the consultation.

Some limitations of the Cancode system should be noted. Coding is time-consuming, and at times, the codes were not always mutually exclusive. For example, the same event could be construed as reflecting more than one function or content category. In particular, the function category is more difficult to code reliably (Dent *et al*, 2005). Inter-rater reliability is lower in these cases. Another limitation is that although 155 patient consultations were analysed, this was based on 5 medical oncologists and 4 radiation oncologists from one of two teaching hospitals in Sydney. Therefore our findings may have limited generalisability to oncologists from other localities. Future studies should aim to increase the size and diversity of their sample of oncologists.

In summary, oncologists in this study varied their consultation style with differing patient characteristics and involvement preferences, and patient outcomes were associated with the oncologist's consultation style. However, there still remained deficiencies in both medical and radiation oncologists adequately addressing the psychosocial and support requirements of cancer patients. The findings presented here provide current and specific evidence that may be used to inform improvements of consultation skills training for oncologists or medical curricula for students. As our findings showed clear differences in the structure of the consultation between medical and radiation oncologists, future studies should examine the moderating effect of this and other doctor/patient characteristics on the relationship between doctors' consultation style and patient outcomes.

## Figures and Tables

**Table 1:** Demographic and disease characteristics of patients (*n*=155)

**Variable**	**Mean (range)**
Age (years)	55.9 (22–82)
	
Length of illness (months)	13 (0–264)
	
*Gender*	(%)
Female	45
Male	55
	
*Education level*	
Below year 10	30.7
Year 10/school certificate	30.1
Year 12/high school certificate	10.5
Tertiary non-university	9.8
University	19.0
	
*Marital status*	
Married or *de facto*	68.6
Single	13.7
Divorced or separated	9.8
Widowed	7.8
	
*Type of cancer*	
Breast	21.0
Colorectal	18.2
Melanoma	15.4
Prostate	14.0
Lymphoma	9.0
Other	22.4
	
*Stage of cancer*	
Local	56.7
Metastasis	43.3
	
*Estimated prognosis*	
Weeks to months	32.6
Years	51.8
Normal life expectancy	15.6

**Table 2:** Average duration (s), s.d. (in parentheses) and the percentage of the total consultation that medical and radiation oncologists spent speaking in each ‘content’ category

	**Medical**		**Radiation**		
**Content category**	**Mean**	**%**	**Mean**	**%**	**F, *P*-value**
History and symptoms	199.0 (96.3)	17.9	88.6 (70.8)	11.0	62.8, <0.0001
Diagnosis	132.8 (123.8)	12.0	79.2 (68.0)	9.8	10.3, <0.01
Prognosis	117.4 (115.8)	10.6	60.0 (51.4)	7.5	14.4, <0.0001
Treatment	500.3 (313.6)	45.1	393.8 (213.1)	48.9	5.8, <0.05
Other medical	50.3 (67.9)	4.5	63.7 (70.3)	7.9	1.4, NS
Psychosocial issues	33.8 (55.9)	3.0	43.3 (58.7)	5.4	1.1, NS
Social support/counselling/stress management	0.5 (2.7)	0.0	7.8 (24.3)	1.0	7.7, <0.01
Social exchange	21.0 (26.1)	1.9	16.3 (21.6)	2.0	1.4, NS
Other/nonspecific	55.1 (56.5)	5.0	52.3 (47.2)	6.5	2

NS=not significant.

**Table 3:** Average duration (s), s.d. (in parentheses) and the percentage of the total consultation of patient and doctor time spent speaking in each ‘function’ category

	**Medical oncologists**		**Radiation oncologists**		**Patients of medical oncologists**		**Patients of radiation oncologists**	
**Function category**	**Mean**	**%**	**Mean**	**%**	**Mean**	**%**	**Mean**	**%**
Reveal intention	82.1 (61.8)	7.4	54.9 (41.6)	6.8	63.6 (80.7)	10.0	62.4 (101.5)	17.0
Advice	105.1 (66.5)	9.5	64.2 (56.0)	8.0	6.3 (12.2)	1.0	3.7 (6.2)	1.0
Questions	181.2 (34.3)	16.3	90.9 (19.4)	11.3	66.0 (19.8)	10.4	50.4 (20.1)	13.7
Label/judge/criticise	0.9 (4.2)	0.1	0.04 (0.3)	0.0	1.7 (5.3)	0.3	1.3 (5.7)	0.3
Express feelings/seek reassurance	2.1 (15.1)	0.2	0.9 (2.8)	0.1	15.8 (31.6)	2.5	9.0 (16.4)	2.5
Inform/educate	664.0 (371.1)	59.8	535.4 (240.2)	66.5	458.8 (250.2)	72.1	225.3 (149.4)	61.3
Actively support	26.3 (38.6)	2.4	24.5 (45.8)	3.0	0.4 (3.1)	0.1	0.3 (1.5)	0.1
Partnership build	35.9 (60.9)	3.2	25.0 (26.0)	3.1	23.6 (35.3)	3.7	15.4 (31.2)	4.2
Check patient understanding	12.5 (15.3)	1.1	9.1 (11.7)	1.1	0.1 (1.0)	0.0	0.03 (0.3)	0.0

**Table 4:** Mean global ratings of consultation style

**Rating scale**	**Medical**	**Radiation**	**F**
Did the doctor appear technically competent and confident? *Competent* *vs* *incompetent*	0.9	1.3	6.7^*^
Did the doctor communicate information to the patient clearly? *Good* *vs* *poor information delivery*	1.7	2.3	4.3^*^
Was the doctor's overall message as positive as possible? *Positive* *vs* *negative*	2.8	3.3	1.8^NS^
Was the consultation patient- or doctor-centred? *Patient-centred* *vs* *doctor-centred*	3.8	5.2	11.7^**^
Was there a sense of the doctor hurrying the consultation? *Very hurried* *vs* *not at all hurried*	7.7	6.6	9.0^*^
In what way did the doctor respond to the patient? *Cold/impersonal* *vs* *warm/caring*	6.3	6.3	<0.01^NS^

^*^P<0.05, ^**^P<0.001, ^NS^Non-significant.

Scores are measured (in centimetres) from the left extreme of each dimension.
